# Association between obesity and hospitalization in mild COVID-19 adult outpatients in Brazil: a prospective cohort study

**DOI:** 10.20945/2359-3997000000486

**Published:** 2022-09-08

**Authors:** Ivaine Tais Sauthier Sartor, Caroline Nespolo de David, Gabriela Heiden Telo, Gabriela Oliveira Zavaglia, Ingrid Rodrigues Fernandes, Luciane Beatriz Kern, Márcia Polese-Bonatto, Thaís Raupp Azevedo, Amanda Paz Santos, Walquiria Aparecida Ferreira de Almeida, Victor Bertollo Gomes Porto, Fernanda Hammes Varela, Marcelo Comerlato Scotta, Regis Goulart Rosa, Renato T. Stein

**Affiliations:** 1 Hospital Moinhos de Vento Responsabilidade Social Porto Alegre RS Brasil Responsabilidade Social, Hospital Moinhos de Vento, Porto Alegre, RS, Brasil; 2 Pontifícia Universidade Católica do Rio Grande do Sul Faculdade de Medicina Porto Alegre RS Brasil Faculdade de Medicina, Pontifícia Universidade Católica do Rio Grande do Sul, Porto Alegre, RS, Brasil; 3 Ministério da Saúde do Brasil Programa Nacional de Imunizações Brasília DF Brasil Coordenação Geral, Programa Nacional de Imunizações, Ministério da Saúde do Brasil, Brasília, DF, Brasil

**Keywords:** Mild COVID-19, noncritical COVID-19, adults, follow-up, outpatients, hospitalization

## Abstract

**Objective:**

To evaluate the association between obesity and hospitalization in mild COVID-19 adult outpatients in Brazil.

**Subjects and methods:**

Adults with signs and symptoms suggestive of acute SARS-CoV-2 infection who sought treatment in two hospital (public and private) emergency departments were prospectively enrolled. Patients with confirmed COVID-19 at inclusion were followed by phone calls at days D7, D14 and D28. Multivariable logistic regression models were employed to explore the association between obesity and other potential predictors for hospitalization.

**Results:**

A total of 1,050 participants were screened, and 297 completed the 28-day follow-up and were diagnosed with COVID-19 by RT-PCR. The median age was 37.2 (IQR 29.7-44.6) years, and 179 (60.0%) were female. The duration of symptoms was 3.0 (IQR 2.0-5.0) days, and 10.0 (IQR 8.0-12.0) was the median number of symptoms at inclusion. Ninety-five (32.0%) individuals had obesity, and 233 (78.5%) had no previous medical conditions. Twenty-three participants (7.7%) required hospitalization during the follow-up period. After adjusting, obesity (BMI ≥ 30.0 kg/m^2^) (OR = 2.69, 95% CI 1.63-4.83, P < 0.001) and older age (OR = 1.05, 95% CI 1.01-1.09, P < 0.001) were significantly associated with higher risks of hospitalization.

**Conclusion:**

Obesity, followed by aging, was the main factor associated with hospital admission for COVID-19 in a young population in a low-middle income country. Our findings highlighted the need to promote additional protection for individuals with obesity, such as vaccination, and to encourage lifestyle changes.

## INTRODUCTION

Coronavirus disease 2019 (COVID-19) has brought large numbers of patients to medical attention within a short period for the care of a previously undescribed illness, challenging health services and providers to deliver time‐sensitive interventions under difficult circumstances. Since the first cases of COVID-19 were documented in China in late December 2019, the epidemiologic focus has primarily been on establishing the severity of disease and outcomes among severe patients (1,2). Older age and some metabolic disorders, such as diabetes, were early predictors of hospital admission and mortality in patients with COVID-19 (3).

The COVID-19 pandemic has occurred when the prevalence of obesity is increasing worldwide. Obesity is a well-established risk factor for metabolic noncommunicable diseases such as type 2 diabetes and hypertension, influencing most major cardiovascular diseases. With the worldwide spread of acute respiratory syndrome coronavirus 2 (SARS-CoV-2), obesity has also emerged as an independent risk factor for severe COVID-19, likely related to the effects of adipose tissue inflammation on the immune system, in addition to all the associated metabolic dysfunction (4). In a recent United States Department of Health and Human Services/Centers for Disease Control and Prevention publication, overweight and obesity were both identified as risk factors for intubation related to COVID-19. In contrast, only obesity was associated with hospitalization and death in an adult cohort of inpatients (5). However, as the risk estimated for severe COVID-19-associated outcomes was measured primarily among adults who received care at a hospital, the current knowledge about the impact of obesity on COVID-19 hospital admission may possibly be overestimated due to selection bias, as most studies evaluated only inpatients.

Few studies have been developed in mild COVID-19 adult outpatients, especially in low-middle income countries (LMICs). Given the uncertainty regarding outcomes in this low-risk outpatient population and considering the exponential rise in the prevalence of obesity and its association with severe forms of the disease, this study aimed to evaluate the association between obesity and hospitalization in mild COVID-19 adult outpatients in Brazil. We also aimed to describe the clinical characteristics of COVID-19 outpatients and identify other predictors of hospitalization.

## SUBJECTS AND METHODS

### Study design and population

This is a prospective cohort study with data collected in two referral hospitals from southern Brazil, one public (*Hospital Restinga e Extremo Sul*) and the other from the private health system (*Hospital Moinhos de Vento*). From 13 May to 14 September 2020, we assessed adult (≥18 years old) outpatients who had a visit to the emergency department (ED) for COVID-19 symptoms. Inclusion criteria were presenting at least one sign or symptom suggestive of COVID-19 (cough, fever, or sore throat) within 14 days of onset of symptoms. We then created a cohort of those with confirmed COVID-19, defined as a positive result on qualitative reverse transcription polymerase chain reaction (RT-PCR) for SARS-CoV-2.

### Study procedures

At baseline, clinical, demographic, comorbidities, and signs or symptoms suggestive of COVID-19 data were collected. All participants underwent bilateral nasopharyngeal and oropharyngeal swab collection for SARS-CoV-2 detection via RT-PCR assay. The samples were analyzed in the Molecular Biology Laboratory at *Hospital Moinhos de Vento*, as described previously (6). Reported weight and height were used to calculate body mass index (BMI). Participants were followed by phone interviews at 7, 14, and 28 days after seeking care due to COVID-19 symptoms (study baseline). Up to ten telephone call attempts were made during three days from D7, D14, or D28. When necessary, text messages were sent to notify them that the researchers were trying to contact them. If there was no success in contacting one of the time points, the questions were asked retroactively on the next call. The questions were asked directly to the participant or, in his absence or incapacity, for a person authorized by him at baseline. The participant was considered lost when it was not possible to make any contact in different day periods (including failure in 10 call attempts or time exceeded considering the 3-day window) within the 28-day follow-up.

During the follow-up interviews, participants were asked about the need for hospital admission, the use of supplemental oxygen, admission to the intensive care unit (ICU), and the use of invasive mechanical ventilation. All data were collected by trained researchers using standardized questionnaires developed for the study in the Research Electronic Data Capture software (REDCap) (7,8).

The outcome of interest was unplanned hospital admission within 28 days. To predict the outcome, demographic and clinical characteristics, such as age at the time of enrollment (years), sex, days of onset of symptoms, racial or ethnic group, active or second hand smoke at home, hospital at inclusion, number of symptoms and number of medical conditions, were considered. To evaluate obesity as a predictor for the outcome of interest, we used BMI, and participants were classified as nonobese (<30.0 kg/m^2^) and obese (≥30.0 kg/m^2^).

### Statistical analyses

Data normality assumptions were verified using the Shapiro-Wilk test for continuous variables, median values and interquartile ranges (IQRs) were calculated, and a two-tailed Mann-Whitney-Wilcoxon test was used. Pearson’s chi-square or Fisher’s exact tests were used to evaluate proportions. Uni- and multivariable logistic regression analyses were performed to assess the predictors of hospitalization considering the follow-up period of 28 days after having a COVID-19 health care visit (study baseline). The association between obesity and hospitalization was assessed using multiple logistic regression models with adjustment for relevant covariates (hospital at inclusion; years of education; number of medical conditions (none, one, two or more); days of onset of symptoms to inclusion; number of symptoms (≤^8^, ^9^–^10^, ^11^–^12^, ≥^13^); and cardiovascular diseases, which included ischemic heart disease, heart failure and hypertension). We performed additional models including covariates that considered risk factors for hospitalization to assess consistency and risk of bias. Odds ratios (ORs) with 95% confidence intervals (CIs) were calculated. All data preprocessing and analyses were performed in R 3.5.0 statistical software (9).

### Ethics

The study was performed following Decree 466/12 (10) of the National Health Council and Good Clinical Practice Guidelines after approval by the *Hospital Moinhos de Vento* Institutional Review Board (IRB) n° 4.637.933. All participants included in this study provided written informed consent.

## RESULTS

**Figure 1:**
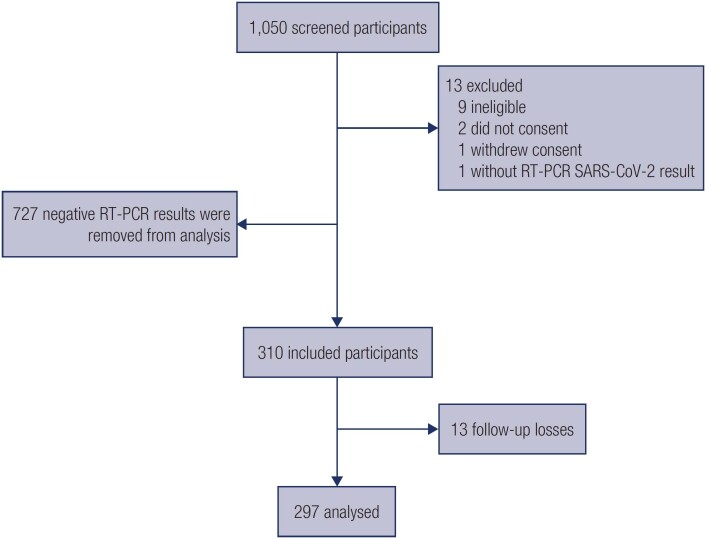
Study flowchart. Thirty-three participants out of 297 answered the questions retroactively since there was no success in contact at that time point. Twelve responsible family members were the respondents.

RT-PCR: reverse transcription polymerase chain reaction; SARS-CoV-2: severe acute respiratory syndrome coronavirus 2.

In this study, 1,050 subjects were screened, and 13 were excluded (nine for not meeting the inclusion criteria, two for not consenting, one for withdrawing consent, and one without SARS-CoV-2 RT-PCR results), and 727 participants with negative RT-PCR results were removed from the analysis. A total of 310 adults with confirmed SARS-CoV-2 were included. Successful 28-day follow-up was obtained for 297 (95.8%) participants included in the final analysis, as shown in Figure 1.

At baseline, the median participants’ age was 37.2 years (IQR, 29.7-44.6). Out of 297 participants, 179 (60.3%) were female, 222 (74.7%) declared themselves Caucasian, and 162 (54.5%) were from the public hospital. The median days of symptoms was 3.0 (IQR, 2.0-5.0), and 10.0 (IQR 8.0-12.0) was the median number of symptoms at inclusion. The most commonly reported symptoms were headache (90.6%), cough (87.2%), malaise (76.4%), and myalgia (76.1%). Few participants were active or second-hand smokers at home (18.5%), and more than half were overweight (107, 36.0%) or with obesity (95, 32.0%), as shown in Supplementary Table 1. Most participants (78.5%) had no previous medical conditions. Hypertension (12.8%), asthma (6.1%) and type 1 or type 2 diabetes (3.0%) were the most frequent baseline comorbidities reported.

At the 28-day follow-up, 23/297 (7.7%) participants required hospitalization. The median BMI of hospitalized participants was 32.4 kg/m^2^ (IQR, 30.2-34.1), whereas for the outpatients, it was 27.7 kg/m^2^ (IQR, 24.3-30.5) (P < 0.001). The median days from onset of symptoms at hospitalization was 8.0 (IQR, 7.0-9.8). During hospitalization, 15/23 (65.2%) participants required only the use of supplemental oxygen, 6/23 (26.1%) were admitted to the ICU, 2/23 (8.7%) used invasive mechanical ventilation, and one participant died (1/23, 4.3%).

**Figure 2:**
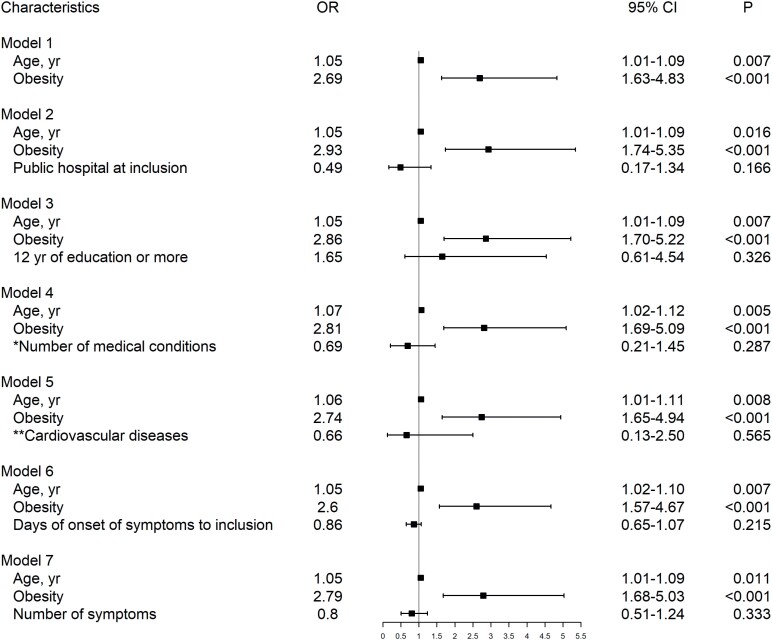
Forestplot of multivariable logistic regression models adjusting age and obesity for the covariates: hospital of inclusion (reference: private hospital at inclusion), years of education (reference: 11 years or less, which corresponds to complete high school education), number of medical conditions (reference: none), cardiovascular diseases (reference: no), days of onset of symptoms (continuous) and number of symptoms (reference: ≤8 symptoms) at enrollment. *Number of medical conditions comprised the sum of cases of hypertension, asthma, diabetes mellitus type 1 or 2, cancer, chronic obstructive pulmonary disease, ischemic heart disease, heart failure, human immunodeficiency virus and previous transplantation (none, one, two or more). **Cardiovascular diseases comprised the presence of ischemic heart disease, heart failure or hypertension. yr: years.

Participants who required hospitalization were significantly older (P = 0.005) and had obesity (P < 0.001) than those who did not. No other differences were observed between groups, as shown in Table 1. Age and obesity were statistically significant in univariable analysis, as shown in Supplementary Table 2. In multivariable logistic regression analysis, age and obesity remained important predictors of hospital admission in our cohort (age: OR = 1.05, 95% CI 1.01-1.09; obesity: OR = 2.69, 95% CI 1.63-4.83). When adjusted for hospital inclusion, years of education, number of medical conditions, cardiovascular diseases, days of onset of symptoms, and number of symptoms at enrollment, age and obesity remained associated with hospitalization, as shown in Figure 2.

**Table 1:** Demographic and clinical characteristics of the analyzed subjects

Characteristics		Participants included in the analysis (n = 297)	Participants without hospitalization (n = 274)	Participants that needed hospitalization (n = 23)	P
Age, median (IQR)		37.2 (29.7-44.6)	36.8 (29.2-44.0)	42.0 (36.5-51.7)	**0.005** *
Female sex, n (%)		179 (60.3)	167 (60.9)	12 (52.2)	0.55†
Active or second hand smoker at home, n (%)		55 (18.5)	51 (18.6)	4 (17.4)	1.00‡
Years of education					
	11 yr or less, n (%)	156 (52.5)	143 (52.2)	13 (56.5)	0.85†
	12 yr or more, n (%)	141 (47.5)	131 (47.8)	10 (43.5)	
Nutritional status					
	Non-obesity (BMI < 30.0 kg/m^2^), n (%)	191 (64.3)	186 (67.9)	5 (21.7)	<0.001†
	Obesity (BMI ≥ 30.0 kg/m^2^), n (%)	95 (32.0)	80 (29.2)	15 (65.2)	
Hospital system at inclusion					
	Private hospital, n (%)	135 (45.5)	124 (45.3)	11 (47.8)	0.98†
	Public hospital, n (%)	162 (54.5)	150 (54.7)	12 (52.2)	
Racial or ethnic group					
	Caucasian, n (%)	222 (74.7)	206 (75.2)	16 (69.6)	0.73†
	Non-Caucasian, n (%)	75 (25.3)	68 (24.8)	7 (30.4)	
Duration of symptoms at inclusion					
	Days, median (IQR)	3.0 (2.0-5.0)	3.0 (2.0-5.0)	3.0 (2.0-3.0)	0.30*
Number of symptoms					
	2-8, n (%)	94 (31.6)	86 (31.4)	8 (34.8)	0.69†
	9-10, n (%)	79 (26.6)	72 (26.3)	7 (30.4)	
	11-12, n (%)	69 (23.2)	66 (24.1)	3 (13.0)	
	≥13, n (%)	55 (18.5)	50 (18.2)	5 (21.7)	
Underlying medical conditions					
	Hypertension, n (%)	38 (12.8)	33 (12.0)	5 (21.7)	0.21‡
	Asthma, n (%)	18 (6.1)	17 (6.2)	1 (4.3)	1.00‡
	Diabetes mellitus, type 1 or 2, n (%)	9 (3.0)	8 (2.9)	1 (4.3)	0.58‡
	Cancer, n (%)	5 (1.7)	5 (1.8)	0 (0.0)	1.00‡
	COPD, n (%)	4 (1.3)	3 (1.1)	1 (4.3)	0.31‡
	Ischemic heart disease, n (%)	3 (1.0)	2 (0.7)	1 (4.3)	0.24‡
	Heart failure, n (%)	1 (0.3)	1 (0.4)	0 (0.0)	1.00‡
	HIV, n (%)	1 (0.3)	1 (0.4)	0 (0.0)	1.00‡
	Previous transplantation, n (%)	1 (0.3)	1 (0.4)	0 (0.0)	1.00‡
	Previous chemotherapy, n (%)	0 (0.0)	0 (0.0)	0 (0.0)	–
	Cardiovascular diseases**, n (%)	38 (12.8)	33 (12.0)	5 (21.7)	0.21‡
Number of medical conditions					
	None, n (%)	233 (78.5)	216 (78.8)	17 (73.9)	0.63†
	One, n (%)	50 (16.8)	46 (16.8)	4 (17.4)	
	Two or more, n (%)	14 (4.7)	12 (4.4)	2 (8.7)	
Hospitalization					
	Yes, n (%)	23 (7.7)	–	–	–
	Days after onset of symptoms to hospitalization, median (IQR)	8.0 (7.0-9.5)	–	8.0 (7.0-9.8)	–
	Days from the ED sought to hospitalization, median (IQR)	5.0 (4.0-7.0)	–	5.0 (4.0-7.0)	–
	Use of supplemental oxygen, n (%)	15 (5.1)	–	15 (65.2)	–
	Admission at ICU, n (%)	6 (2.0)	–	6 (26.1)	–
	Use of invasive mechanical ventilation, n (%)	2 (0.7)	–	2 (8.7)	–
	Deaths, n (%)	1 (0.3)	–	1 (4.3)	–

* Mann-Whitney-Wilcoxon test.

† Pearson’s Chi-squared test.

‡ Fisher’s exact test.

** Cardiovascular diseases comprised: ischemic heart disease, heart failure and hypertension.

IQR: interquartile range; yr: years; BMI: body mass index; COPD: chronic obstructive pulmonary disease; ED: emergency department; ICU: intensive care unit.

Among the 15 participants with obesity and COVID-19 admitted to the hospital within 28 days of follow-up, the median age was 42.0 years (IQR, 37.2-51.7), 9 (60.0%) were male, and 8 (53.3%) were admitted to private hospitals. Four (26.6%) participants reported underlying medical conditions (hypertension, diabetes mellitus, asthma, chronic obstructive pulmonary disease or ischemic heart disease). The median days of onset of symptoms to hospitalization was 8.0 (IQR, 7.0-9.0), and the median days from the ED sought to hospitalization was 5.5 (IQR, 4.0-7.0). Ten participants (66.7%) required supplemental oxygen, 4 (26.7%) were admitted to the ICU, one needed invasive mechanical ventilation (6.7%), and one participant died (6.7%).

## DISCUSSION

Obesity is a recognized risk factor associated with worse outcomes related to COVID-19; however, most previous studies were carried out in a population of patients already admitted to the hospital or at a high admission rate due to the disease (11). In this study, we evaluated the risk of hospital admissions in two institutions, one public and one private, in southern Brazil through an outpatient cohort of individuals enrolled early in the course of the disease. For an adult low-risk population, our results identified that obesity was the main predictor of hospitalization for COVID-19, with an approximately 3-fold increase in the risk of hospital admission compared to patients without obesity.

The world is still struggling to fight the COVID-19 pandemic. According to the World Health Organization (WHO), the mortality rate from COVID-19 is 0.5%-1.0% (12), while the hospitalization rate for the disease is 5.0%-15.0%. Based on currently available information, the Centers for Disease Control and Prevention has identified severe obesity as an important risk factor for worse prognosis and higher mortality in patients with COVID-19. In contrast, any degree of obesity has been associated with poor COVID-19 prognosis. Individuals with obesity live in a low-grade state of chronic inflammation resulting in metabolic and immune dysregulation, which is supposed to be the leading cause of a worse prognosis during SARS-CoV-2 infection (13). In our study, 7.4% of our sample required hospital admission, similar to the literature data. Most participants were overweight/with obesity at baseline, but only obesity predicted the main outcome. In a meta-analysis of 75 studies (14), individuals with obesity had a 46.0% higher risk for being COVID-19 positive, a 113.0% higher risk of being hospitalized, and a 48.0% increased mortality. Many mechanisms jointly may explain this impact; although the effect of obesity on the immune system remains not entirely established, it appears to have an effect independent of coexisting risk factors. The data from the literature also identify a dose-response relationship between BMI and worse COVID-19 outcomes (5).

In addition to metabolic factors that seem to impact the severity of COVID-19, advanced age has been identified as one of the major factors associated with worse outcomes in the pandemic, being one of the most important predictors of hospital admission (11). In our sample, the mean age of participants was younger than that in most studies in the literature. However, we also identified an increased risk of hospitalization for COVID-19 with each increased year of age. Concerns about our data include the fact that the predictors of higher risk for COVID-19 involve populations, especially those in need of care by the health system (5). Elderly individuals with obesity are possibly the most compromised by social distancing due to the pandemic, resulting in worse health habits, such as physical inactivity and unhealthy eating habits, in addition to increased emotional distress. This could further aggravate the risk related to COVID-19 for this population, unless governmental and health measures, such as vaccination and strategies to increase consumption of healthier foods, stimulate physical activity and promote mental health (14), aimed at the health of this population, start to be prioritized.

Our study has limitations. Our analysis was based on a convenience sample. Moreover, our participants represent a single geographic region of a heterogeneous country, such as Brazil. However, the worldwide prevalence of obesity is a public health concern, and our findings are in line with previous reports (15–18). As another point to address, the two centers included in this study represent different populations, public and private, which may characterize different clusters and limit the interpretation of our results for the sample as a whole. Additionally, most of our data represent self-reports and may be biased. Nevertheless, we believe that our results add to the literature as they represent an outpatient prospective cohort of adults with a low risk for complications related to COVID-19, identifying important risk predictors for this population. Moreover, a similar impact of obesity was consistently found despite adjusting for several possible confounders.

In conclusion, our data identified that obesity, in addition to age, was the most critical risk factor predicting hospital admission for COVID-19. These results highlight the importance of health support strategies aimed at this population to promote additional protection, such as vaccination, and encourage lifestyle changes.

## Reviewer

A version of this study is in: medRxiv preprint doi: https://doi.org/10.1101/2021.08.04.21261538. The copyright holder for this preprint is the author/funder, who has granted medRxiv a license to display the preprint in perpetuity. It is made available under a CC-BY-NC-ND 4.0 International license.

## Appendix

**Supplementary Table 1:** Demographic and clinical characteristics of the total number of included individuals and the characterization of those who did not complete the follow-up period

Characteristics		Participants included in the study (n = 310)	Follow-up losses (n = 13)
Age, median (IQR)		37.4 (29.8-45.0)	44.2 (33.2-47.0)
Female sex, n (%)		186 (60.0)	7 (53.8)
Active or second hand smoker at home, n (%)		55 (17.7)	NI
Years of education			
	11 yr or less, n (%)	156 (50.3)	NI
	12 yr or more, n (%)	141 (45.5)	NI
Nutritional status			
	Non-obesity (BMI < 30.0 kg/m^2^), n (%)	201 (64.8)	10 (76.9)
	Obesity (BMI ≥ 30.0 kg/m^2^), n (%)	98 (31.6)	3 (23.1)
Nutritional status II			
	Underweight (BMI < 18.5 kg/m^2^), n (%)	2 (0.6)	1 (7.7)
	Normal weight (BMI ≥ 18.5 and ≤ 24.9 kg/m^2^), n (%)	87 (28.1)	4 (30.8)
	Overweight (BMI > 24.9 and ≤ 29.9 kg/m^2^), n (%)	112 (36.1)	5 (38.5)
	Obesity (BMI ≥ 30.0 kg/m^2^), n (%)	98 (31.6)	3 (23.1)
Hospital system at inclusion			
	Private hospital, n (%)	137 (44.2)	2 (15.4)
	Public hospital, n (%)	173 (55.8)	11 (84.6)
Racial or ethnic group			
	Caucasian, n (%)	222 (71.6)	NI
	Non-Caucasian, n (%)	75 (24.2)	NI
Duration of symptoms at inclusion			
	Days, median (IQR)	3.0 (2.0-5.0)	3.0 (2.0-5.0)
Number of symptoms			
	2-8, n (%)	95 (30.6)	1 (7.7)
	9-10, n (%)	82 (26.5)	3 (23.1)
	11-12, n (%)	74 (23.9)	5 (38.5)
	≥13, n (%)	59 (19.0)	4 (30.8)
Underlying medical conditions			
	Hypertension, n (%)	39 (12.6)	1 (7.7)
	Asthma, n (%)	19 (6.1)	1 (7.7)
	Diabetes mellitus, type 1 or 2, n (%)	11 (3.5)	2 (15.4)
	Cancer, n (%)	5 (1.6)	0 (0.0)
	COPD, n (%)	4 (1.3)	0 (0.0)
	Ischemic heart disease, n (%)	3 (1.0)	0 (0.0)
	Heart failure, n (%)	1 (0.3)	0 (0.0)
	HIV, n (%)	1 (0.3)	0 (0.0)
	Previous transplantation, n (%)	1 (0.3)	0 (0.0)
	Previous chemotherapy, n (%)	0 (0.0)	0 (0.0)
	Cardiovascular diseases**, n (%)	39 (12.6)	1 (7.7)
Number of medical conditions			
	None, n (%)	243 (78.4)	10 (76.9)
	One, n (%)	52 (16.8)	2 (15.4)
	Two or more, n (%)	15 (4.8)	1 (7.7)
Hospitalization			
	Yes, n (%)	23 (7.4)	–
	Days after onset of symptoms to hospitalization, median (IQR)	8.0 (7.0-9.5)	–
	Days from the ED sought to hospitalization, median (IQR)	5.0 (4.0-7.0)	–
	Use of supplemental oxygen, n (%)	15 (4.8)	–
	Admission at ICU, n (%)	6 (1.9)	–
	Use of invasive mechanical ventilation, n (%)	2 (0.6)	–
	Deaths, n (%)	1 (0.3)	–

** Cardiovascular diseases comprised: ischemic heart disease, heart failure and hypertension. NI: not informed.

IQR: interquartile range; yr: years; BMI: body mass index; COPD: chronic obstructive pulmonary disease; ED: emergency department; ICU: intensive care unit.

## Appendix

**Supplementary Table 2:** Results from univariable logistic regression analysis

Variable	OR (95%CI)	P
Age, years (continuous)	**1.05 (1.02-1.09)**	**0.002**
Days of onset of symptoms to inclusion (continuous)	0.89 (0.71-1.07)	0.240
Years of education (reference: 11 years of education)	0.84 (0.35-1.97)	0.690
Hospital system at inclusion (reference: private hospital)	0.90 (0.38-2.15)	0.812
Nutritional status (reference: non-obesity)	**2.64 (1.61-4.69)**	**<0.001**
Number of medical conditions (reference: none)	1.25 (0.58-2.38)	0.525
Number of symptoms (reference: 2-8)	0.94 (0.63-1.38)	0.755
Cardiovascular diseases (reference: no)	2.03 (0.64-5.48)	0.189

## References

[1] Grasselli G, Zangrillo A, Zanella A, Antonelli M, Cabrini L, Castelli A (2020). Baseline Characteristics and Outcomes of 1591 Patients Infected With SARS-CoV-2 Admitted to ICUs of the Lombardy Region, Italy. JAMA.

[2] Altonen BL, Arreglado TM, Leroux O, Murray-Ramcharan M, Engdahl R (2020). Characteristics, comorbidities and survival analysis of young adults hospitalized with COVID-19 in New York City. PLoS One.

[3] Berlin DA, Gulick RM, Martinez FJ (2020). Severe Covid-19. N Engl J Med.

[4] Huang Y, Lu Y, Huang YM, Wang M, Ling W, Sui Y (2020). Obesity in patients with COVID-19: a systematic review and meta-analysis. Metabolism.

[5] Kompaniyets L, Goodman AB, Belay B, Freedman DS, Sucosky MS, Lange SJ (2021). Body Mass Index and Risk for COVID-19-Related Hospitalization, Intensive Care Unit Admission, Invasive Mechanical Ventilation, and Death – United States, March-December 2020. MMWR Morb Mortal Wkly Rep.

[6] Polese-Bonatto M, Sartor ITS, Varela FH, Gianinni GLT, Azevedo TR, Kern LB (2021). Children have similar RT-PCR cycle threshold for SARS-CoV-2 in comparison with adults. medRxiv.

[7] Harris PA, Taylor R, Thielke R, Payne J, Gonzalez N, Conde JG (2009). Research electronic data capture (REDCap)--a metadata-driven methodology and workflow process for providing translational research informatics support. J Biomed Inform.

[8] Harris PA, Taylor R, Minor BL, Elliott V, Fernandez M, O’Neal L (2019). The REDCap consortium: Building an international community of software platform partners. J Biomed Inform.

[9] R The R Project for Statistical Computing [Internet].

[10] Ministério da Saúde [Internet] Resolução nº 466, de 12 de dezembro de 2012.

[11] Petrilli CM, Jones SA, Yang J, Rajagopalan H, O’Donnell L, Chernyak Y (2020). Factors associated with hospital admission and critical illness among 5279 people with coronavirus disease 2019 in New York City: prospective cohort study. BMJ.

[12] World Health Organization Estimating mortality from COVID-19: Scientific brief, 4 August 2020 [Internet].

[13] Korakas E, Ikonomidis I, Kousathana F, Balampanis K, Kountouri A, Raptis A (2020). Obesity and COVID-19: immune and metabolic derangement as a possible link to adverse clinical outcomes. Am J Physiol Endocrinol Metab.

[14] Popkin BM, Du S, Green WD, Beck MA, Algaith T, Herbst CH (2020). Individuals with obesity and COVID-19: A global perspective on the epidemiology and biological relationships. Obes Rev.

[15] Sales-Peres SHC, de Azevedo-Silva LJ, Bonato RCS, Sales-Peres MC, Pinto ACS, Santiago JF (2020). Coronavirus (SARS-CoV-2) and the risk of obesity for critically illness and ICU admitted: Meta-analysis of the epidemiological evidence. Obes Res Clin Pract.

[16] Simonnet A, Chetboun M, Poissy J, Raverdy V, Noulette J, Duhamel A (2020). High Prevalence of Obesity in Severe Acute Respiratory Syndrome Coronavirus-2 (SARS-CoV-2) Requiring Invasive Mechanical Ventilation. Obesity (Silver Spring).

[17] Nakeshbandi M, Maini R, Daniel P, Rosengarten S, Parmar P, Wilson C (2020). The impact of obesity on COVID-19 complications: a retrospective cohort study. Int J Obes.

[18] Kang Z, Luo S, Gui Y, Zhou H, Zhang Z, Tian C (2020). Obesity is a potential risk factor contributing to clinical manifestations of COVID-19. Int J Obes.

